# A Rare Case of Lung Hypoplasia in a 1-Year-Old Girl

**DOI:** 10.7759/cureus.31529

**Published:** 2022-11-15

**Authors:** Atef A Rashed, Esraa Sendi, Ghaida Alzahrani, Sadeem Bukhari, Deemah H Bukhari

**Affiliations:** 1 Pediatrics, Maternity and Children's Hospital, Makkah, SAU; 2 Medicine and Surgery, Medical College of Umm Al-Qura University, Makkah, SAU; 3 Otolaryngology - Head and Neck Surgery, Maternity and Children's Hospital, Makkah, SAU

**Keywords:** congenital lung malformations, congenital lung abnormalities, lung abnormalities, lung hypoplasia, pulmonary hypoplasia, congenital anomalies

## Abstract

Pulmonary hypoplasia is a rare form of the congenital disorder that leads to lung underdevelopment. It is more common in children and infrequently noticed in adulthood. While congenital lung abnormalities are frequently discovered in the early years of life, these conditions are also often detected incidentally following routine radiographic imaging and investigations. We report the case of a 1-year-old girl who presented to the emergency department with a three-day history of cough, shortness of breath, fever, and respiratory distress. Investigations revealed right lung hypoplasia, left lung hyperinflation, and an invisible pulmonary artery and vein. A diagnosis of right lung hypoplasia as an incidental finding with associated anomalies was made. She was admitted to the pediatric ward and received supportive care and empirical antibiotics. She was then discharged in a good condition with supportive management for follow-up. This case report aims to describe a rare condition occurring in children with various clinical pictures and presentations to aid future early detection to achieve better diagnostic outcomes.

## Introduction

Pulmonary or lung hypoplasia is among a wide range of phenomena presented by underdeveloped tissues of the lung [1]. This relatively rare medical condition is characterized by the incomplete growth of the lungs that consequently affects a child’s development. Further, inadequate gas exchange and respiratory impairment can arise from a reduction in the number of airways and alveoli [2]. Pulmonary hypoplasia may reflect the presence of congenital malformation, including other systems such as the cardiovascular system and central nervous system [1]. It can be classified as primary (idiopathic, abnormal intrinsic lung disease) and secondary to other anomalies. The etiology of primary pulmonary hypoplasia is not fully understood. Many factors could be involved in this malformation, including hereditary, environmental, maternal, and nutritional factors [3]. Congenital lung malformations may be apparent in the newborn period but are also diagnosed incidentally on routine imaging and investigations [4].

We report the case of a 1-year-old girl who presented with cough, shortness of breath, fever, and respiratory distress and was diagnosed with right lung hypoplasia with accompanying anomalies as an incidental finding.

## Case presentation

**Figure 1:**
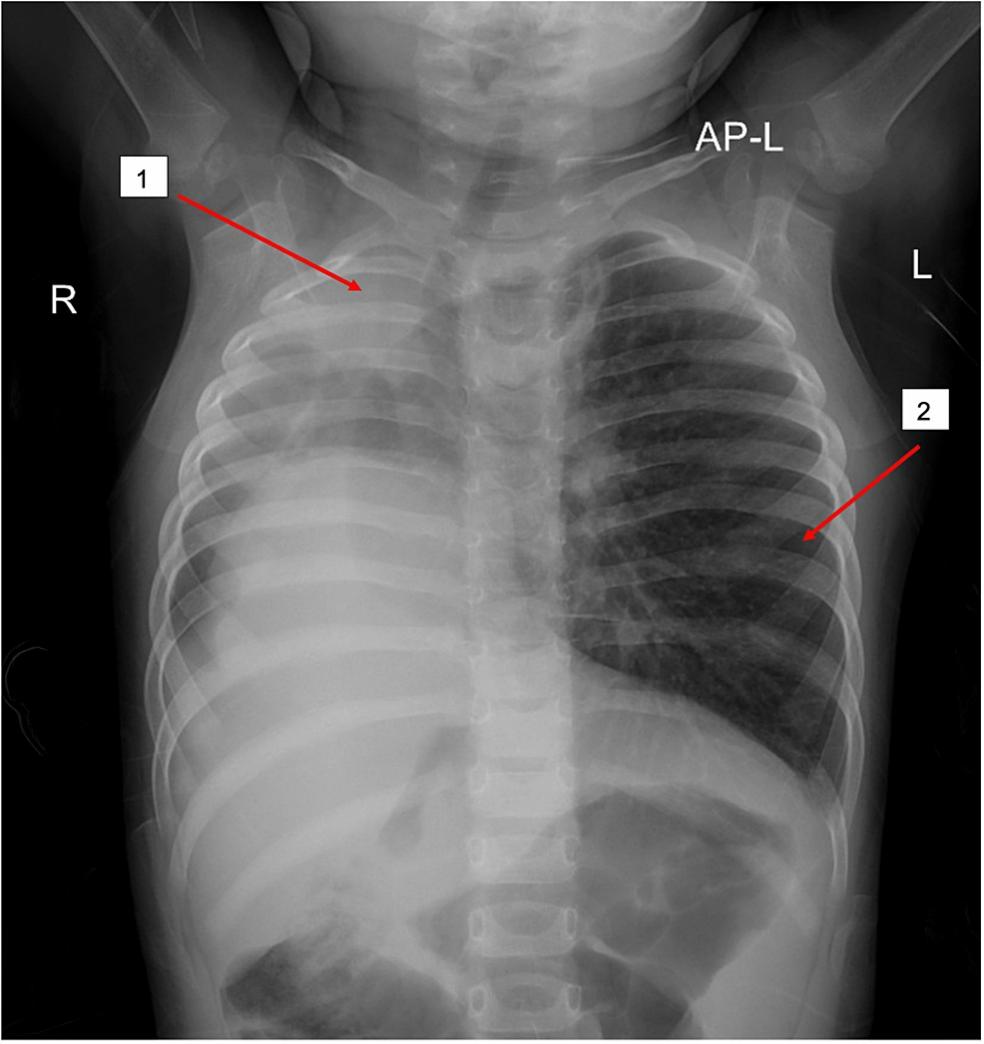
The anterior-posterior view of CXR showed right upper lobe opacity with lung hypoplasia (1) and a left hyperinflated lung (2). CXR: chest X-ray

A 1-year-old full-term girl presented to the emergency department (ED) with a three-day history of cough, shortness of breath, and fever. Past medical and surgical histories were unremarkable. And, there is no previous history of neonatal intensive care unit (NICU) admission or respiratory symptoms. On general physical examination, the patient was ill and conscious. Vital signs were measured as follows: temperature (T): 38.2°C, heart rate (HR): 120 beats/minute, respiratory rate (RR): 45 breaths/minute, blood pressure (BP): 96/52 mmHg, and oxygen saturation: 88% on room air and 98% on 2 L nasal cannula. The patient had signs of respiratory distress in form of nasal flaring and subcostal retraction. Chest examination showed decreased air entry and an apical beat on the right side of the lung with crepitation, while the left side showed good air entry with no added sound. Asymmetrical chest movement was not noted. Other systemic examinations were unremarkable. Laboratory investigations revealed no abnormalities. A chest X-ray (CXR) revealed the following incidental findings: right upper lobe opacity with lung hypoplasia and a left hyperinflated lung. In addition, there is cardiac dextroposition as the heart is displaced to the right side due to lung hypoplasia (Figure 1).

**Figure 2:**
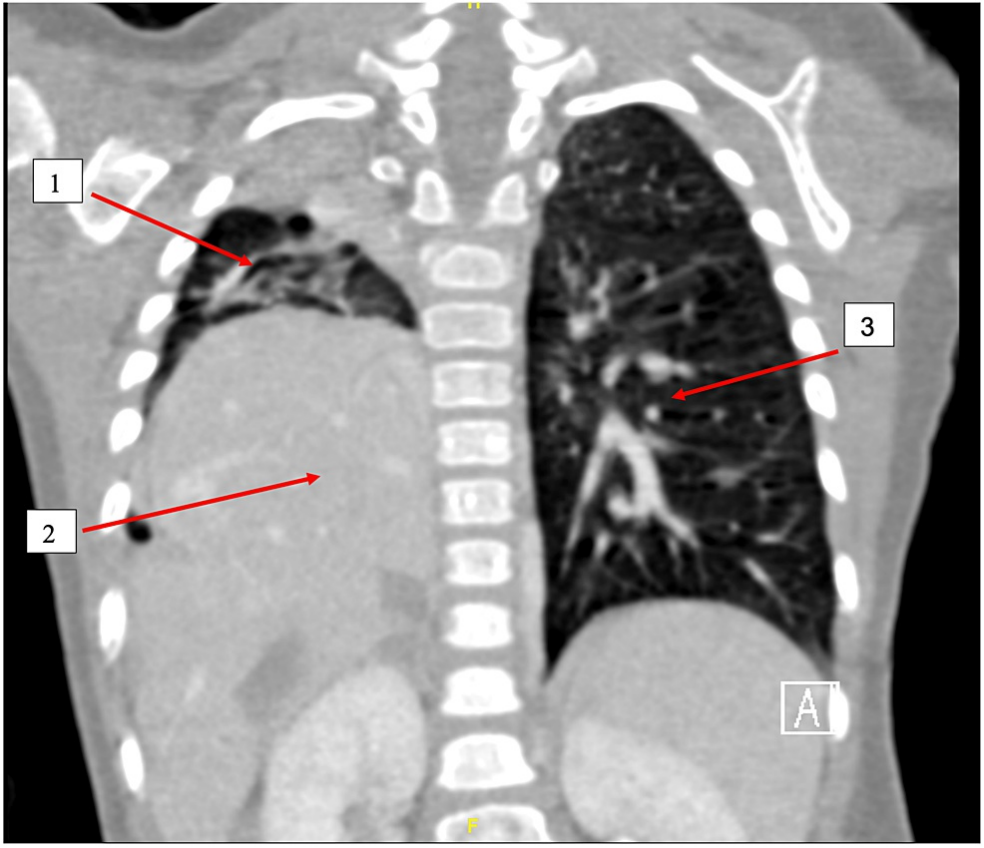
The coronal view of chest CTA showed hypoplastic right lung (1), dextroposition of the heart due to right lung hypoplasia (2), and left lung opacification and bronchiectatic changes (3). CTA: computed tomography angiography

She was admitted with a case of pneumonia for further workup and investigations. On admission, her growth parameters were normal for her age. Moreover, chest computed tomography angiography (CTA) and an echocardiogram (ECHO) were ordered to establish the diagnosis. The CTA report revealed the following results: an absent right pulmonary artery, dilated main and left pulmonary arteries (14-15 mm), absent right pulmonary veins, a hypoplastic right lung with complete opacification and bronchiectatic changes, compensatory hyperinflation of the left lung, and herniation/focal diaphragmatic eventration with an upward displacement of the right liver lobe into the right posterior hemithorax (Figure 2).

The ECHO results showed the dextroposition of the heart with normal myocardial function, levocardia with situs solitus, normal systemic venous connections, left pulmonary vein drain to the left atrium, and invisible right pulmonary veins. Also, it showed that the atrioventricular and ventricular-atrio were concordant, the size of the intra-arterial septum and interventricular septum was normal, an invisible right pulmonary artery, and the absence of patent ductus arteriosus. In addition, a bronchoscopy was performed and the report confirmed the presence of right upper bronchus atresia.

The diagnosis of right lung hypoplasia was made according to the patient’s clinical history and radiological findings. During her hospital stay, the patient received supportive management, including oxygen for the first two days, intravenous (IV) fluids, salbutamol, and normal saline nasal drops. She was further prescribed antibiotics (ceftriaxone and clindamycin) for pneumonia treatment. Eventually, she was discharged in good condition after significant clinical improvement. The future care plan included follow-up CTA and symptomatic treatment.

## Discussion

Pulmonary hypoplasia is a rare condition [1]. Congenital lung abnormalities may cause symptoms in the neonatal period or early childhood; however, they can also be discovered by coincidence during routine surveillance if the individual has not demonstrated any disease-related symptoms [5]. The clinical manifestation and extent of respiratory dysfunction are determined by the accompanying abnormalities and severity of hypoplasia [1]. The incidence of pulmonary hypoplasia is estimated as 0.9 to 1.1 per 1000 live births for both primary and secondary pulmonary hypoplasia. It is more prevalent among children and rarely diagnosed in adults [3].

We reported the case of a 1-year-old girl who presented to the ED with cough, shortness of breath, fever, and respiratory distress for three days. A CXR showed right lung hypoplasia with a hyperinflated left lung. The CTA and ECHO used to identify associated cardiac anomalies revealed the absence of the right pulmonary artery and right pulmonary vein.

Studies have found that children can experience respiratory distress at birth or an Apgar score requiring supplemental oxygen or mechanical ventilation; nevertheless, newborns with normal Apgar scores have also been documented [6,7]. Additionally, some patients have a silent childhood and the disease is manifested later, as in our patient. Patients with aplasia and hypoplasia may experience recurrent infection, tachypnea, and dyspnea. Bronchial secretions may accumulate with secondary infections [8].

In pulmonary hypoplasia, the contralateral lung is hyperinflated and enlarged, with a larger number of alveoli. Similarly, the CXR of our patient showed a hyperinflated and enlarged left lung [8]. Unilateral pulmonary artery agenesis with lung hypoplasia is a type of lung developmental defect. It is considered to be uncommon to have pulmonary artery agenesis on the same side. A previous study described the case of a 15-year-old male patient who presented with shortness of breath and chest pain and was ultimately diagnosed with left pulmonary artery agenesis and left lung hypoplasia [6]. Similarly, we confirmed the presence of right lung hypoplasia with the absence of the right pulmonary artery.

Chest X-ray is helpful in the diagnosis of pneumonia. However, sputum culture and gram stain, and blood culture are usually negative in pneumonia cases [9]. The radiological findings of our patient confirmed the presence of lung opacification and infiltration. According to radiological findings and the patient clinical signs of respiratory distress, the decision was made to start ceftriaxone as a broad-spectrum antibiotic and clindamycin to cover anaerobes. Currently, no treatment is available for pulmonary hypoplasia [1]. The primary method of management should consist of follow-up and symptomatic treatment. In conditions with pulmonary hypoplasia, operative intervention ought only to be sought in cases in which cardiac and vascular abnormalities are present, persistent episodes of hemoptysis, or chronic chest infections and bronchiectasis [6]. Major pulmonary vascular abnormalities include interruption of the main pulmonary artery or its absence, the emergence of the left pulmonary artery in the right pulmonary artery, pulmonary venous drainage abnormalities, and pulmonary arteriovenous malformations (PAVMs) [10].

In general, a lot of cases of lung hypoplasia are associated with vascular abnormalities but the literature did not specify specific types of vascular abnormalities that require surgical intervention as we know. Surgical intervention depends on many aspects as overall clinical status, radiological investigation, and the severity of vascular and cardiac abnormalities and associated symptoms. We have looked into this further, however, the decision of continuing the conservative management only or surgical intervention is determined after considering all aspects [10,11,12]. A multidisciplinary team is now following up with the patient which consists of a thoracic surgeon, a pediatrician, and a radiologist. The management approach plan is mainly conservative and the possibility of a pulmonary right lung lobectomy is depend on many aspects during follow-up. In this case, the patient received appropriate supportive care and was discharged in a healthy condition.

## Conclusions

Pediatric pulmonary hypoplasia can be a diagnostic and therapeutic challenge. This case report aims to raise awareness of a rare condition in the pediatric population that may manifest with a variety of clinical presentations in order to promote early recognition for diagnosis and optimize the potential outcomes. We discussed the case of a 1-year-old girl who presented to the ED with a first-time episode of respiratory distress and was accidently discovered to have right pulmonary hypoplasia after performing a CXR. Many conditions are associated with pulmonary hypoplasia and determining the underlying cause is crucial to decrease mortality. While no strict diagnostic criteria have thus far been established for pulmonary hypoplasia, the radiological or postpartum histological investigation might aid in the diagnosis.
